# Phytochemical Profiling, Antioxidant, Antimicrobial and Cholinesterase Inhibitory Effects of Essential Oils Isolated from the Leaves of *Artemisia scoparia* and *Artemisia absinthium*

**DOI:** 10.3390/ph15101221

**Published:** 2022-10-01

**Authors:** Farman Ali Khan, Nasir Mehmood Khan, Shujaat Ahmad, Nasruddin, Riffat Aziz, Ihsan Ullah, Mazen Almehmadi, Mamdouh Allahyani, Ahad Amer Alsaiari, Abdulelah Aljuaid

**Affiliations:** 1 Department of Chemistry, Shaheed Benazir Bhutto University, Sheringal, Dir Upper 18000, Khyber Pakhtunkhwa, Pakistan; 2 Department of Agriculture, Shaheed Benazir Bhutto University, Sheringal, Dir Upper 18000, Khyber Pakhtunkhwa, Pakistan; 3 Department of Pharmacy, Shaheed Benazir Bhutto University, Sheringal, Dir Upper 18000, Khyber Pakhtunkhwa, Pakistan; 4 Department of Environmental Sciences, Shaheed Benazir Bhutto University, Sheringal, Dir Upper 18000, Khyber Pakhtunkhwa, Pakistan; 5 Department of Clinical Laboratory Sciences, College of Applied Medical Sciences, Taif University, P.O. Box 11099, Taif 21944, Saudi Arabia

**Keywords:** *Artemisia scoparia*, *Artemisia absinthium*, essential oils (EOs), EOAS, EOAA, phytochemical profiling, GC-MS, antioxidant effects, DPPH, ABTS, antibacterial, antifungal activities

## Abstract

The current studies were focused on the phytochemical profiling of two local wild *Artemisia* species, *Artemisia scoparia* and *Artemisia absinthium* leaves’ essential oils, extracted via the hydro distillation method along with evaluation of their antioxidant as well as antimicrobial effects. The constituents of EOs were identified using a combined gas chromatography-mass spectrometric (GC-MS) technique. A total of 25 compounds in *A. scoparia* essential oil (EOAS) were identified, and 14 compounds with percentage abundance of >1% were tabulated, the major being tocopherol derivatives (47.55%). A total of nine compounds in *Artemisia absinthium* essential oil (EOAA) were enlisted (% age > 1%), the majority being oleic acid derivatives (41.45%). Strong antioxidant effects were pronounced by the EOAS in DPPH (IC_50_ = 285 ± 0.82 µg/mL) and in ABTS (IC_50_ = 295 ± 0.32 µg/mL) free radical scavenging assays. Both the EOs remained potent in inhibiting the growth of bacterial species; *Escherichia coli* (55–70%) and *Shigella flexneri* (60–75%) however remained moderately effective against *Bacillus subtilis* as well as *Staphylococcus aureus*. Both EOAS and EOAA strongly inhibited the growth of the tested fungal species, especially *Aspergillus* species (up to 70%). The oils showed anti-cholinesterase potential by inhibiting both Acetylcholinesterase (AChE; IC_50_ = 30 ± 0.04 µg/mL (EOAS), 32 ± 0.05 µg/mL (EOAA) and Butyrylcholinesterase (BChE; IC_50_ = 34 ± 0.07 µg/mL (EOAS), 36 ± 0.03 µg/mL (EOAA). In conclusion, the essential oils of *A. scoparia* and *A. absinthium* are promising antioxidant, antimicrobial and anticholinergic agents with a different phytochemical composition herein reported for the first time.

## 1. Introduction

Essential oils (EOs) are the volatile constituents mainly found in the flowers and fresh leaves of plants, often stored in the epidermal tissues [1]. EOs have been used as flavoring factors in the food industries, perfumes, beverages, cosmetics, pharmaceuticals and nutraceuticals, etc. [2]. Chemically, EOs are mixtures of low molecular weight organic compounds such as mono and sesquiterpenoids, phenylpropenes, isothiocyanates, etc. [3] and possess a broad spectrum of potent biological effects including antifungal, antibacterial, anti-diabetic, anticancer, antiviral and, most recently, have been considered effective against COVID-19 respiratory infections [4,5,6]. Approximately 3000 different EOs have been obtained from more than 2000 plant species with production of 40,000–60,000 tons per year and revenue generation of about 7.5 billion US$ in 2018 with a 9% increase until 2026 [7].

*Artemisia* is a diversified genus of flowering plants, comprising approximately 500 species related to the daisy family of Asteraceae [8]. *Artemisia* contains various herbs, plants and shrubs, which are famous due to its antimalarial constituent, artemisinin [9] and rich in essential oils [10]. Two of the species including *Artemisia scoparia* (Local name = Tarkha) and *Artemisia absinthium* (Local name = Zoon) have been found in abundance in the Dir districts, KP, Pakistan [11,12]. The different types of *Artemisia* are locally used to treat inflammation, jaundice, malaria, tumors and various microbial infections and their essential oils possess strong antispasmodic, anti-hepatotoxic and anti-jaundice activities [13]. Some of the major constituents of *Artemisia* essential oils are *caryophyllene oxide, borneol, camphor, thujane, thujanol, myrtenol, linalyl acetate, limonene* and *pinene* etc. [13]. It has been observed that various factors such as genetic mutation, plant nutrition, geographic location, seasonal variation, surrounding climate, growth, and maturity affect the constitution of EOs of *Artemisia*. Moreover, the production of EOs also depends upon the nature of plant materials and methods of extraction. The current study is proposed to investigate the essential oils composition, antioxidant, antimicrobial and cholinesterase potential of two local *Artemisia* species, *A. scoparia* and *A. absinthium* essential oils.

## 2. Results and Discussion

### 2.1. GC-MS Analysis of the Artemisia scoparia Essential Oil (EOAS)

**Figure 1:**
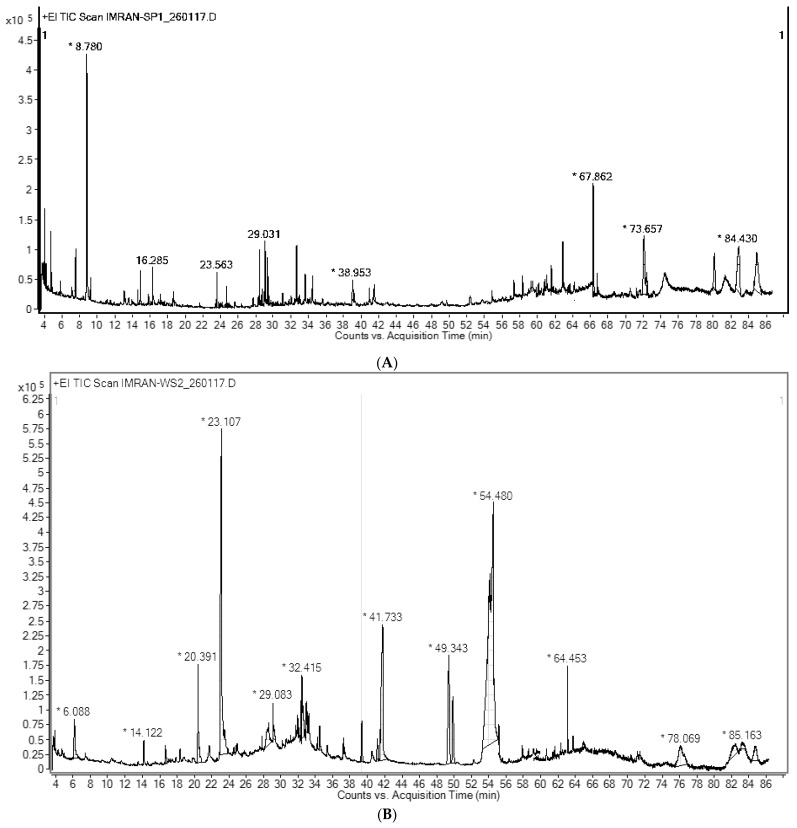
(**A**) Total Ion Chromatogram (TIC) of EOAS; (**B**) Total Ion Chromatogram (TIC) of EOAA. * = identification matched >50% with database.

A total of 31 peaks were recorded for EOAS by gas chromatography (GC) in which 25 compounds were analyzed and 14 were quantified (% > 1%) (Figure 1A). The resolutions were obtained by comparison with the system GC-MS library, the mainlib and riplib databases, as well as estimated through a non-polar retention index (*n*-alkane scale), available literature as well as NIST online library. These findings are summarized in Table 1. The most abundant compound found was 7-Hydroxy-bicyclo[3.3.1] non-2-en-9-one (17.05%), while Tocopherol-β-D-mannoside was detected in 16.21% quantity. The other major constituents obtained were D-α-Tocopherol (13.65%), (+)-α-Tocopherol acetate (12.54%), and 18,19-Secoyohimban-19-oic acid was in an 8.24% ratio. α-Tocopherol acetate was found to exist in 5.15%, while the other constituents were present in very minor amounts. Overall, the essential oil displayed a strange composition as its major components were derivatives of tocopherol, a strong antioxidant. These tocopherol derivatives have never been detected in such quantities earlier nor have been reported, to the best of our knowledge. Similarly, amine derivatives such as 18,19-Secoyohimban-19-oic acid has been found for the first time in the essential oil of this species. The majority of terpenoids found were sesquiterpenenes and their oxygenated derivatives, while the monoterpenoids and their oxygenated derivatives were found in relatively small quantities. The presence of chromene derivatives suggested the possibility of flavone type constituents. According to previous studies, the essential oils of A. scoparia contained 1,8-cineole, p-cyamene, limonene, β-myrecene, camphor, β-pinene, terpenine, α, β-thujone etc. [14].

### 2.2. GC-MS Analysis of the Artemisia absinthium Essential Oil (EOAA)

The nine compounds having a percentage abundance of >1% were identified and quantified by gas chromatography-mass spectral (GC-MS) analysis (Figure 1B). These findings are summarized in Table 2. The most abundant compound found was Oleic acid (41.45%), while 2-Furancarboxaldehyde, 5-(hydroxymethyl) was detected in 14.24% quantity. The other major constituents identified included 9,12-Octadecadienoic acid (Z,Z)-, methyl ester (9.01%), Estra-1,3,5(10)-trien-17β-ol (8.75%), 9,9-Dimethoxybicyclo[3.3.1]nona-2,4-dione (7.74%) and *N-(1-methoxycarbonyl-1-methylethyl)-4-methyl-2-aza-1,3-dioxane* was in a 5.5% ratio, while the remaining were detected in very minute quantities. These types of constituents have been reported for the first time from this species, especially the amine derivative N-(1-methoxycarbonyl-1-methylethyl)-4-methyl-2-aza-1,3-dioxane.

### 2.3. Antioxidant Activity

The antioxidant potential of both EOAS and EOAA were measured using both DPPH and ABTS scavenging assays in a dose dependent manner. The EOAS of A. Scoparia was extremely active in inhibiting DPPH, 85 ± 0.91% and ABTS, 83 ± 0.43% at the dose of 500 µg/mL (Table 3). The IC_50_ values were obtained as 285 ± 0.82 µg/mL against DPPH and 295 ± 0.32 µg/mL against ABTS, respectively. However, at the same concentration, the EOAA of *A. absinthium* showed low antioxidant effect against DPPH (60 ± 0.31%) and ABTS (57 ± 0.47%), respectively. The potent antioxidant effect of the A. scoparia EO in both the assays could be attributed to the presence of huge quantities of tocopherol derivatives as well as sesquiterpene derivatives. The A. Scoparia essential oils previously showed strong antioxidant effects in scavenging both hydroxyl ions as well as hydrogen peroxide at doses of 25–200 µg/mL [15]. Similarly, the huge quantities of oleic acid derivatives could be responsible for the antioxidant effects of *A. absinthium* EO since higher IC_50_ values have been reported earlier in its antioxidant activities with different phytochemical profiles (DPPH= 29.72 mg/mL and ABTS = 38.83 mg/mL) [16]. In a recent study, the EO obtained from A. aragonesis was an effective antioxidant against DPPH (IC_50_ 340 μg/mL); however, our results are in contradiction with the reported concentrations where the results in this study demonstrated IC_50_ values of 285 ± 0.82 µg/mL (EOAS against DPPH) and IC_50_ values of 295 ± 0.32 µg/mL (EOAS, against ABTS), respectively [17]. Similarly, the EO obtained from A. Judaica showed strong antioxidant activities by enhancing the catalytic functions of superoxide dismutase and catalase enzymes [18].

Reactive oxygen species (ROS) can cause serious oxidative harmful effects on macromolecules, especially the biomolecules. These are the chemically reactive ions/radicals produced as byproducts from the primary metabolism and include singlet oxygen, hydrogen peroxide, hydroxyl radicals or superoxide anion radicals. An excess of such ROS in bodies leads to the damage of the enzymatic processes as well as alters the function of various proteins, lipids, and even DNA which result in cerebral dementia type diseases such as Parkinson’s disease (PD), Alzheimer’s disease (AD), diabetes mellitus (DM) as well as cancer development [19,20,21,22]. The balancing of “oxidative stress” induced to the body by ROS could be achieved by using antioxidants as medications or supplements. The EO of various Artemisia species have previously been explored for their antioxidant effects and often correlated to the presence of oxygenated mono or sesquiterpenoids [23]. Another study revealed that the presence of eucalyptol, linalool as well as β-myrecene in *A. absinthium* essential oils could impart potent antioxidant and herbicidal effects [24]. It has also been observed that an increase in the antioxidant effect usually follows a dose dependent manner, especially in case of essential oils [25]. Our results reveal the presence of tocopherol derivatives in EOAS. Tocopherol and its acetate derivatives have been used as reference drugs for measuring the antioxidant effects in various studies [26], and thus EOAS proved to be best candidate for use as an antioxidant in further studies. Oleic acid and its derivatives have previously been used for the measurement of antioxidant effects in various studies, [27] hence, EOAA could be a possible new antioxidant agent.

### 2.4. Antibacterial Activities

**Figure 2:**
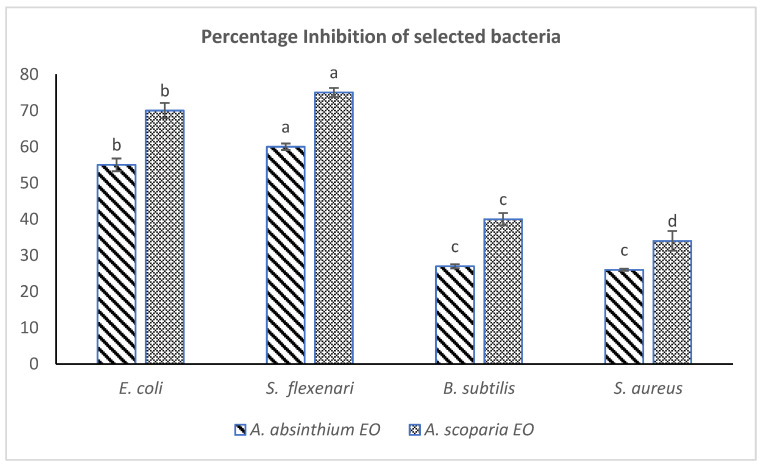
Comparative antibacterial effect (% inhibition) of EOAS and EOAA (dose of 500 µg/mL DMSO). One same letter shows no significant difference.

Both of the plants EOs remained effective in imparting strong antibacterial effects against the tested bacteria, especially the Gram-negative EOAS inhibited *E. coli* (70%) and *S. flexneri* 75%, while EOAA inhibited *E. coli* 55% and *S. flexneri* 60% respectively at the same doses (500 µg/mL). The percentage inhibition calculated from the measurements of zones of inhibitions have been provided in Figure 2. The EOs of *A. scoparia* have earlier been reported to possess strong antibacterial effects against 15 oral bacteria while various species of *Salmonella* and *Bacillus* were found suspectable to the oils of *A. absinthium*. [28,29]. It has been observed that EO of many *Artemisia* species exert strong antibacterial effects. The EO of *A. abrotanum* and *A. afra* has been effective against *Staphylococcus aureus* and other pathogenic organisms [30,31]. The oils of *A. persica* and *A. asiatica* showed strong antibacterial effects, especially against *E. coli* [32,33,34]. Studies on *A. absinthium* EOs suggested strong antibacterial activities (MIC ranged from <800 µ/mL) against some species [16]. Moreover, the presence of phenolic/alcoholic groups in the essential oil components (*thymol*, *carvacrol*) have been shown to possess strong antibacterial effects especially on *Bacillus* species as well as *Campylobacter* species such as *C. jejuni* and *C. coli* [35,36]. Most recently, the EO obtained from *A. aragonesis* through hydro distillation was effective in inhibiting various pathogenic bacteria such as *E. coli*, *B. subtills* and *S. aureus* with MIC < 7 µg/mL, much strongly than EOAS/EOAA. This may be due to the presence of different phytochemicals (*Camphor*, 24%) in its EOs [17].

These may be used as alternative antibiotics in animals. The formulations of small quantities of such essential oils have been proven to inhibit the production of *Streptococcus* species, *E. coli,* and have been successfully been formulated into skin care creams against dermatitis [37]. Moreover, the EO of many other species of Dir-Kohistan have also been investigated for their phytochemical profiles as well as biological activities [38].

Recently, plant essential oils are getting attention as promising antibacterial agents due to low toxicity, smaller dose and resistance of pathogenic bacteria to the existing antibiotics. The evidence of pharmacological activities, especially against antimicrobial action, has been well established for essential oil components (especially the major ones) that possess hydrophilic functionalities in their lipophilic skeletons [39]. Moreover, various essential oils have been considered good antimicrobial agents towards Gram-positive rather than Gram-negative bacteria. Gram-negative bacteria constitute cell envelopes of the outer lipopolysaccharide layer over their thin cell walls composed of the peptidoglycan layer. This structural feature acts as a barrier towards the penetration of hydrophobic compounds and limits their diffusion into bacterium cytoplasms [40]. In addition, the Lipid A component of their complex lipopolysaccharide layer produces toxicity towards the human immune defense system [41]. Our findings suggest that both EOAS and EOAA can inhibit the growth of Gram-negative bacteria, probably with different modes of actions. One of the most probable pathways includes either the ease of entrance of the smaller constituents of EOs through the outer membranes of Gram-negative bacteria or the incorporation of these constituents with bacterial enzyme systems [42].

It has been observed that the EO of *Artemisia* from their leaves differed in their phytochemicals in comparison with literature. e.g., EO of *A. firgida*, *A. cana*, *A. tridentata*. The oils overall showed a weak pharmacological effect (anti-inflammatory) in activating the human neutrophils; however, the relatively higher effect was showed by *A. tridentata* due to the presence of *farnesene* (smaller amounts) [43]. A recent study of various *Artemisia* species suggested the presence of sufficient amounts of oxygenated monoterpenoids that imparted potential insecticidal activities (LD_50_ = 17–52 μg/mL), a possible therapeutic application for environmentally friendly pesticides [44].

### 2.5. Antifungal Activity

**Figure 3:**
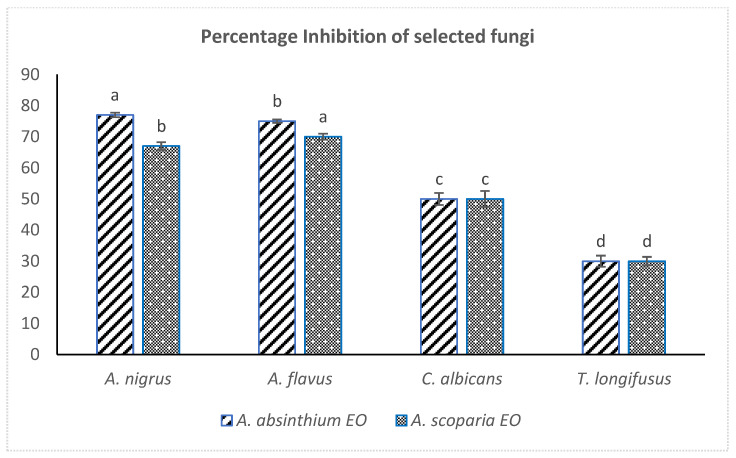
Comparative antifungal effects (% inhibition) of EOAS and EOAA (dose of 500 µg/mL DMSO). One same letter shows no significant difference.

Both the EOAS and EOAA showed strong antifungal effects against the *Aspergillus* species (70% inhibition of *Aspergillus niger* and 67% *Aspergillus flavus*), while they remained ineffective against *Candida albicans* and *Trichophyton longifusus*. The linear growth and control linear growth were effectively measured for calculating the percentage inhibition of EOs against the tested fungal strains (Figure 3). The results are consistent with the reported antifungal effects of EOs of *A. judaica, A. absintium* and *A. biennis* against *Aspergillus niger*, however, no reports have been documented on the antifungal activity of *A. scoparia* EO against *A. flavus* so far [45]. Strong antifungal effects have been documented in the literature for *Artemisia* essential oils along with their cytotoxic, antihepatotoxic and antimalarial activities [46,47]. The EO obtained from *A. Judaica* was potent against the *Candida* and *Aspergillus* species [17].

Fungi are mostly tough targets for the essential oils to inhibit at micro and macro levels. The pathogenic fungi including *Aspergillus* and *Candida* species are notorious and harmful for large populations, causing serious infections, especially in those with immune deficiencies, hence the need for greater attention [48]. Various human pathogenic fungi including *Candida albicans* show susceptibility to the essential oils of *Apiaceae* plants, cinnamon, lemongrass, clove oil etc. [49,50]. The oil is rich in sesquiterpenoids such as *bisabolol*, inhibits the growth of *Aspergillus niger, A. flavus* and *Candida* species, and thus confirms our findings [8].

### 2.6. Acetylcholinesterase (AChE) and Butyrylcholinesterase (BChE) Inhibition Activities

The results against AChE and BChE suggested that both the EOs successfully inhibited these enzymes in vitro by showing IC_50_ values of 30 ± 0.04 µg/mL (EOAS), 32 ± 0.05 µg/mL (EOAA) against AChE and 34 ± 0.07 µg/mL (EOAS), 36 ± 0.03 µg/mL (EOAA) against BChE respectively (Table 4). More promising results have been noted in BChE inhibition where the IC_50_ values of EOAS (34 ± 0.07 µg/mL) and EOAA (36 ± 0.03 µg/mL) were close to the IC_50_ of allanzanthane (IC_50_ 24.32 ± 0.04).

These promising results are in contrast to the previous results obtained for the *Artemisia* species, where the IC_50_ ranged from 100 to 200 µg/mL [51]. This potent effect could be attributed to the different chemical profiling of our EOs, especially the presence of tocopherol derivatives in EOAS as well as the occurrence of oleic acid in the EOAA. Tocopherol is a reversible inhibitor of AChE and has kinetically been estimated to slowly bind to human enzymes with 2 min of residence time on target. Moreover, the molecular docking studies suggest that tocopherol non-specifically interacts with the alkylene chain of enzymes without producing any conformational changes [15]. *A. scoparia* has also demonstrated a well-established anti-Alzheimer’s disease (AD) effect by lowering β-amyloid precursor protein levels through cleaving related enzymes as well as suppressing the expression of phosphorylated glycogen synthase kinases in vivo, thus making this plant a good candidate against AD and related dementias [52].

EOAA was enriched (41%) in oleic acid contents, which is a remarkable selective inhibitor of BChE in earlier reports. It has been observed that oleic acid binds to different allostatic sites of enzyme by interaction to OH and NH_3_ functional groups at cystine 319 and tyrosine 320 and show some non-competitive reversible inhibition [53]. The literature suggests that various plant extracts containing smaller amounts of oleic acid have shown promising anti-cholinesterase effects [54,55]. However, the major effect has been observed against BChE in many cases. Our study suggests that EOAA strongly inhibits both the enzymes which render a potential use of *Artemisia* based formulations against AD and related diseases.

## 3. Materials and Methods

### 3.1. Identification of Plant Material and Collection

The plants *A. scoparia* and *A. absinthium* were identified by plant taxonomist, Prof. Dr. Ali Hazrat, University of Malakand, Lower Dir, KPK, Pakistan. Both the plants are wild and not being cultivated. The voucher specimens (accession number A-039; A-040) were deposited in the herbarium of Natural Product Laboratory, Department of Chemistry, SBBU, Sheringal. The collection of aerial parts (leaves and shoots) of *A. scoparia* was made from Dog Dara (altitude 5367 feet), Sheringal, Dir (Upper), Pakistan (35.33° N latitude; 71.97° E longitude) while that of *A. absinthium* were collected from Munda, Dir lower (35.56° N latitude; 72.20° E longitude; 8100 feet altitude).

### 3.2. Isolation of Essential Oils (EOs)

The oils were extracted from the fresh leaves of both *Artemisia* species through hydro-distillation methods using a Clevenger apparatus [56]. Fresh mature leaves (250 g) of each plant were separated, sliced and combined with distilled water in a round bottom flask (1 L) fixed with a glass column (60 cm length) and connected with a condenser. Each combination was boiled and the oils from the hydro-distillate were decanted from the snout of the condenser. The procedure was repeated four times for each species with fresh leaves. *A. scoparia* yielded pale yellow colored oil (0.2% *w*/*w*), while *A. absinthium* yielded a light greenish color oil (0.35% *w*/*w*). The collected oils were dried over anhydrous magnesium sulphate and kept in the dark at 4 °C for further analysis.

### 3.3. Gas Chromatography and Mass Spectrometry (GC/MS) Analysis

The phytochemical profile of essential oil samples was determined through GC/MS analysis. An Agilent USB=393752 GC (Agilent Technologies, Palo Alto, CA, USA) connected to an Agilent HP 5973 Mass Spectrometer (mass selective detector) in electron impact (EI, 70 eV) mode was used to obtain GC/MS data. The GC was equipped with a HHP-5MS 5% phenylmethylsiloxane column (capillary column, 30 m × 0.25 mm × 0.25 μm film thickness, Restek, PaloAlto, CA, USA) having an FID detector. Helium gas was used as carrier gas (flow rate 1 mL/min) while a dynamic oven temperature range was used. The initial temperature was retained at 70 °C for 1 min followed by a 6 °C/min increase up to 180 °C. The temperature was again retained for 5 min at 180 °C and then increased at a rate of 5 °C up to 280 °C and retained for 20 min. The samples were injected in a splitless mood (1/1000 in pentane, *v*/*v*). The scan range was 50 *m/z* to 800 *m/z.* The individual compounds were identified through comparative analysis with their retention times and spectral pattern on the same conditions from our database, NIST libraries as well as Kovat retention indexes (relative calculation with n-alkane series on same columns).

### 3.4. 1,1-diphenyl-2-picryl-hydrazyl (DPPH) Free Radical Scavenging Assay

DPPH was purchased from Sigma Aldrich (Burlington, MA, USA), while all the other solvents used were of analytical grade. The assays of EOAS and EOAA were carried according to the method described in the literature with slight modifications [57]. Briefly, the EOs stock solution was prepared by the addition of 500 μg EO (each) with 1 mL methanol while 0.075 mM DPPH solution was prepared in methanol. To initiate the reaction, 0.1 mL of EOs solution was added into 3.9 mL of DPPH solution, homogenized at 2500 rpm for 2 min to remove any cloudy substance, and the absorbance was measured at 517 nm (t = 0) on a BMS spectrophotometer (VIS-1100, New York, USA). The mixtures were then incubated in the dark for 30 min at room temperature and then their absorbance was again measured at 517 nm. The control sample was prepared by mixing 0.1 mL methanol in a 3.9 mL DPPH solution. IC_50_ values for the mixture samples were obtained by serial dilution and individual measurements. The following formula was applied in calculating the percent absorbance.

(1) $$\%\;\mathrm{DPPH}\;\mathrm{free}\;\mathrm{radical}\;\mathrm{scavenging}=\frac{\mathrm{Absorbance}\;\mathrm{of}\;\mathrm{control}-\mathrm{absorbance}\;\mathrm{of}\;\mathrm{sample}}{\mathrm{Absorbance}\;\mathrm{of}\;\mathrm{control}}\times 100$$

### 3.5. 2,2′-azino-bis(3-ethylbenzothiazoline-6-sulphonic acid (ABTS) Method

An ABTS assay was performed for further measurements of the antioxidant potential of the essential oils [58]. According to this method, a 10 mL ABTS substrate was mixed with 25 μL of 3% hydrogen peroxide to obtain the standard curve (A). In the next step, 10 μL of trolox (reference standard) was added in wells with the addition of 20 μL of myoglobin working solution to obtain a trolox reference curve (B). In the next step, 10 μL test sample + 20 μL of myoglobin working solution were added to the wells from which the test samples (C) were collected. Finally, the reactions were started with the addition of 150 μL ABTS substrate solution to each well (having A, B, C solutions), incubated for 5 min, and, after that, 100 μL of stop solution was added. The endpoint was monitored at 405 nm using a plate reader. The concentration of EO used for this assay was 500 µg/mL in DMSO. The following formula was used for measurement of ABTS effect.

(2) $$\%\;\mathrm{ABTS}\;\mathrm{free}\;\mathrm{radical}\;\mathrm{scavenging}=\;\frac{\mathrm{Absorbance}\;\mathrm{of}\;\mathrm{control}-\mathrm{absorbance}\;\mathrm{of}\;\mathrm{sample}}{\mathrm{Absorbance}\;\mathrm{of}\;\mathrm{control}}\times 100$$

### 3.6. Antibacterial Assay

The agar disc diffusion procedure was used for the measurement of antibacterial activity of the essential oils against the selected strains e.g., *Escherichia coli* (ATCC 15224), *Bacillus subtilis* (ATCC 6663), *Staphylococcus aureus* (ATCC 29213) and *Shigella flexneri* (ATCC 14028). The stock solutions for the sample were prepared in 500 µg/mL concentrations in DMSO, while 100 and 200 μL of each dilution were added to the concerned wells (control contained = 100 and 200 μL DMSO). The reference drug used was clarithromycin. The diameters for the zone of inhibition were measured in mm, converted into a percentage while the MIC of the antibiotic was measured using standard methods [59].

### 3.7. Fungicidal Assay

The standard method, “agar dilution”, was performed to measure the antifungal activity of the essential oils [60]. The strain used in this assay included *Aspergillus flavus* (ATTC 32611), *Aspergillus niger*, clinically isolated, obtained from the microbiology lab, Agha Khan University Hospital, Karachi, Pakistan), *Candida albicans* (ATTC 2091) and *Trichophyton longifusus* (ATTC 22397). The 500 µg/mL concentration of the sample was dissolved in DMSO (sterile). The linear growth of the fungal strains was monitored visually after seven days (incubation in humid conditions at 37 °C). Media nourishment growth was detected by measuring linear growth (mm), and growth inhibition was calculated with reference to the negative control (also presented in %), (miconazole as reference drug).

### 3.8. Cholinesterase Inhibitory Assay and IC_50_ Values Determination

A modified spectroscopic method for the assessment of cholinergic potential of essential oils was adopted [61]. The enzymes used in this assay were acetylcholinesterase (AChE, EC 3.1.1.7) and butyrylcholinesterase (BChE, EC. 3.1.1.8), the substrates used were, acetylcholine iodide and butyrylcholine chloride, while the standard used was Allanzanthane and Galantamine. 5-5′-dithiobis[2-nitrobenzoic acid] (DTNB) acted as the colorimetric reagent. All the chemicals used were of analytical grade. Accordingly, 40 µL of AChE/BChE and 40 µL of test solution were mixed in 880 µL of sodium phosphate buffer (pH = 8) while 0.2 mM DTNB was also added to each sample solution. The reaction was started by the addition of 40 µL of acetylcholine iodide or butyrylcholine chloride to each sample followed by incubation for 15 min and then monitoring the yellow color of solutions at 412 nm due to the formation of 5-thio-2-nitrobenzene as the product of hydrolysis of acetylthiocholine and butyryl thiocholine. All of the experiments were performed in replicas of five assays. The IC_50_ values were determined from various concentrations using the serial dilution method. A double beam BMS spectrophotometer (USA) was used for λ_max_ measurements.

### 3.9. Statistical Analysis

The data were evaluated statistically by a Student’s *t*-test. *p* values less than 0.05 and 0.01 were taken as significant. Values are expressed as mean ± S.E.M.

## 4. Conclusions

The current studies have been carried out to identify the constituents of essential oils (EOs) obtained from the leaves of *Artemisia scoparia* and *A. absinthium* as well as their antioxidant and antimicrobial effects. The oils were obtained by the hydro-distillation method. A total of 14 compounds were detected in *A. scoparia* with a major portion composed of tocopherol derivatives, while oleic acid derivatives were the dominant constituents in *A. absinthium*. Strong antioxidant effects were pronounced by the EOs of *A. scoparia;* however, *A. absinthium* showed lower antioxidant effects. Both of the EOs potentially inhibited the growth of the tested Gram-negative bacteria, however, remained low potent against Gram-positive. Both the oils were active in inhibiting the *Aspergillus* species. The oils showed pronounced cholinergic potential in inhibiting the tested enzymes, acetylcholinesterase and butyrylcholinesterase in vitro. Our findings reveal that the essential oils of *A. scoparia* and *A. absinthium* are promising antioxidant, antimicrobial and cholinergic agents, even if devoid of aroma components.

## Figures and Tables

**Table 1:** Quantification assessment of major phytochemicals in EOAS.

S. No.	Area Sum%	Compound Name	RT ^a^	NIST #	DB ID * = Mainlib ** = Replib
1	2.66	2-Ethenyl-bicyclo[2.1.1]hexan-2-ol	8.924	221,372	50,226 *
2	17.05	7-Hydroxy-bicyclo[3.3.1]non-2-en-9-one	8.930	193,435	41,812 *
3	1.65	Decane	14.867	114,147	21,679 *
4	1.37	2,6,11,15-tetramethyl-hexadecane, (Crocetane)	23.559	114,255	22,806 *
5	1.22	1-Isopropyl-4,7-dimethyl-1,3,4,5,6,8a-hexahydro-4a(2H)-naphthalenol (cubenol)	27.628	140,968	80,121 *
6	2.11	4,5,9,10-dehydro-isolongifolene	28.380	151,550	105,281 *
7	2.99	2,4-bis(1,1-dimethylethyl)-phenol	29.031	228,966	140,190 *
8	1.73	β-Himachalenoxide	30.996	140,216	74,268 *
9	1.6	Estra-1,3,5(10)-trien-17β-ol	41.42	254,855	7219 *
10	8.24	18,19-Secoyohimban-19-oic acid	67.862	48,433	21,782 *
11	13.65	d-α-Tocopherol	73.657	151,382	122,577 *
12	5.15	dl-α-Tocopherol	81.652	230,590	123,023
13	12.54	(+)-α-Tocopherol acetate	84.43	154,596	28,227 **
14	16.21	Tocopherol-β-D-mannoside	86.48	156,682	123,458 *
		**Total area = 91.83%**			

^a^ = RI calculated. Comparison with literature from **#** NIST, mainlib and riplib databases. Estimated non-polar retention index (n-alkane scale).

**Table 2:** GC and GC-MS analysis of *A. absinthium* essential oil (AAEO).

S. No.	Area Sum%	Compound Name	RT ^a^	NIST #	DB ID * = Mainlib ** = Replib
1	7.74	9,9-Dimethoxybicyclo[3.3.1]nona-2,4-dione	14.122	106,223	7209 *
2	2.85	2,4-Dihydroxy-2,5-dimethyl-3(2H)-furan-3-one	20.391	281,424	9854 *
3	5.55	N-(1-Methoxycarbonyl-1-methylethyl)-4-methyl-2-aza-1,3-dioxane	20.471	146,417	105,697 *
4	14.24	5-(hydroxymethyl)-2-Furancarboxaldehyde	23.227	231,276	60,271 *
5	2.2	Guanosine	28.555	212,407	2164 **
6	2.69	3,4-diethyl-1,1′-Biphenyl	32.375	62,426	150,478 *
7	8.75	Estra-1,3,5(10)-trien-17β-ol	41.791	254,855	7219 *
8	9.01	9,12-Octadecadienoic acid (Z,Z)-, methyl ester	49.253	333,205	28,886 *
9	41.45	Oleic Acid	54.227	228,066	4483 **
		**Total area = 94.48%**			

^a^ = RI calculated. Comparison with literature from # NIST, mainlib and riplib databases. Estimated non-polar retention index (n-alkane scale).

**Table 3:** DPPH and ABTS free radical scavenging effects of EOAS and EOAA.

Concentration	% Inhibition (500 µg/mL)	IC_50_ Value
**DPPH**		
**EOAS**	85 ± 0.91	285 ± 0.82 µg/mL
**EOAA**	60 ± 0.31	416 ± 0.45 µg/mL
**Standard drug ^a^**	100	55 ± 0.56 µg/mL
**ABTS**		
**EOAS**	83 ± 0.43	295 ± 0.32 µg/mL
**EOAA**	57 ± 0.47	433 ± 0.65 µg/mL
**Standard drug ^b^**	100	1.0 ± 0.63 µg/mL

^a^ = Ascorbic acid in DPPH assay; ^b^ = Trolox for ABTS assay.

**Table 4:** Cholinesterase inhibition data of EOAS and EOAA.

Sample	AChE ± SEM ^a^ (IC_50_ in µg/mL)	BChE ± SEM (IC_50_ in µg/mL)
EOAS	30 ± 0.04	34 ± 0.07
EOAA	32 ± 0.05	36 ± 0.03
Allanzanthane ^b^	6.38 ± 0.04	24.32 ± 0.04
Galantamine ^b^	8.22 ± 0.03	17.03 ± 0.05

^a^ = Standard Error Mean of five assays; ^b^ = positive controls; data shown are values from triplicate experiments.

## Data Availability

Data is contained within the article.
